# An Audit on Did Not Attend (DNA) Rate in the Learning Disability Outpatient Mental Health Setting, BCHFT

**DOI:** 10.1192/bjo.2025.10588

**Published:** 2025-06-20

**Authors:** Ayesha Fazal ur Rehman, Joseph Vella

**Affiliations:** 1 Black Country Healthcare NHS Foundation Trust, Sandwell, United Kingdom

## Abstract

**Aims:** Service users not attending outpatient clinics without prior notification are considered as DNA. We intended to identify the factors contributing to DNA in ID patients, with a view to mitigate the risks and safeguarding concerns associated with it and also to assess staff compliance with the Trust’s DNA policy.

**Methods:** Trust DNA policy states that “Ring patient after DNA, to check if the patient was aware of the appointment, and discuss the barriers of non attendance and then document accordingly.”

Using this policy as standard, we retrospectively evaluated the electronic patient notes who did not attend their appointments over the period of 2 months. Data regarding demographic details, diagnosis, neuropsychiatric comorbidities, social circumstances, treatment modalities and post DNA actions were collected.

**Results:** Out of the total 117 appointments over 2-month period, 35 (29%) patients didn’t attend scheduled appointment. 69% of these patients were males, 47% live with their family, 38% in residential settings, 13% in supported living and 3% live alone. The severity (mild, moderate or severe) of intellectual disability was distributed approximately equally comprising about one-third each, with 3% needing diagnostic formulation. 51% have co-morbid autism, 14% have epilepsy and another 14% have depression. Only 3% have comorbid psychotic illness. 91% are on regular psychotropics, 6% on as-required medication and 3% are receiving psychosocial interventions only.

In terms of post DNA actions, staff contacted 34% via telephone after the DNA, however no details aligned with the policy were being documented. No contact made for 57% of the patients, and for 9% there was no documentation on electronic progress notes. 83% were offered another appointment, 17% got discharged back to the care of GP.

**Conclusion:** Non-attendance at appointments is most significantly influenced by male gender, living with family and having neuropsychiatric comorbidity, which appeared as the dominant contributing factors. Compliance with the trust’s policy is below standard, recommendations were suggested to adapt DNA policy for this patient group and to increase awareness among divisional staff during the Induction programme.

